# School absenteeism is linked to household food insecurity in school catchment areas in Southern Nevada

**DOI:** 10.1017/S136898002100063X

**Published:** 2021-10

**Authors:** Courtney Coughenour, Brooke Conway Kleven, Maxim Gakh, Haroon Stephen, Lung-Chang Chien, Brian Labus, Regis Whaley

**Affiliations:** 1 Department of Environmental and Occupational Health, School of Public Health, University of Nevada, Las Vegas (UNLV), Mail Stop 3063, 4700 S. Maryland Parkway, Suite 335, Las Vegas, NV 89119, USA; 2 Department of Epidemiology and Biostatistics, School of Public Health, University of Nevada, Las Vegas (UNLV), Las Vegas, USA; 3 Department of Civil and Environmental Engineering and Construction, Howard R. Hughes College of Engineering, University of Nevada, Las Vegas (UNLV), Las Vegas, USA; 4 Three Square Food Bank, Las Vegas, USA

**Keywords:** Food insecurity, Food security, School absenteeism, School attendance

## Abstract

**Objectives:**

Food security and school attendance are both important for health, well-being and academic performance of children and adolescents. However, their intersection remains underexamined, especially in the USA. The current study considered the association between elementary school-level absenteeism and household food insecurity.

**Design:**

The current study linked school-level absenteeism and household food insecurity rates using geographic information system mapping and applied the tobit regression model to examine their association.

**Setting:**

The Clark County, Nevada, public school district – the fifth largest in the USA and in a state with disproportionate food insecurity and chronic school absenteeism rates.

**Participants:**

Data consisted of school-level absenteeism rates from 185 elementary schools and census tract-level household food insecurity rates.

**Results:**

Average daily attendance rates were lower for schools with catchment areas that had higher average household food insecurity (FI), decreasing by −0·0232 % per 1 % increase in FI rate (*P*-value = 0·022). They were also significantly associated with most absenteeism risk factors. Average daily attendance rate was negatively associated with Free and Reduced Lunch eligibility percentage (−0·010 per 1 % increase in FI, *P*-value < 0·001) and Individualized Education Program participation percentage (−0·039 % per 1 % increase in FI, *P*-value = 0·033), but positively associated with parent–teacher conference participation rate (0·006 % per 1 % increase in FI, *P*-value = 0·025) and white student percentage (0·011 % per 1 % increase in FI, *P*-value = 0·022).

**Conclusions:**

The current study suggests a link between household food insecurity and elementary school-level absenteeism. Understanding this link is important for policy and practice because schools are frequent settings for food insecurity mitigation interventions.

Food security plays a key role in child health and well-being^(1)^. The U.S. Department of Agriculture defines food insecurity as having “a household-level economic and social condition of limited or uncertain access to adequate food”^(2)^. Households with children are more likely to be food insecure; in 2018, 13·9 % of households with children were food insecure compared with 9·9 % of households without children^(3)^. Although poverty status is a strong indicator for household food insecurity, still 6·5 % of households with children at or above 185 % of the federal poverty level remained food insecure^(3)^. Other factors associated with increased risk for food insecurity include parent and child access to health insurance and health care, low levels of parental education, single-parent-headed households, maternal age and living in a Black non-Hispanic or Hispanic-headed household^(4–6)^. Inadequate nutrition, as well as behavioural and psychosocial outcomes associated with these risk factors, may adversely impact children’s physical and emotional well-being.

It has been shown by several studies that household-level food insecurity “even at marginal levels, is associated with children’s behavioural, academic, and emotional problems from infancy to adolescence across western industrialized countries”^(1,7)^. In their systematic review, Shankar and colleagues concluded that among school-age children, there is an association between food insecurity and “impaired academic performance, increased hyperactivity, inattention, aggressive behaviour, missing school, borderline emotional problems, less adaptive interpersonal relations, self-control and approaches to learning, more internalising and externalising behaviours and greater likelihood of having seen a psychologist”^(1)^. Specifically, academic performance in reading and mathematics has been shown to be impaired in children who live or lived in food insecure homes during early childhood^(8)^. Furthermore, Murphy and colleagues suggest that even intermittent experiences of food insufficiency and hunger are associated with poor behavioural and academic functioning in low-income children, including increased rates of school absenteeism and tardiness^(9)^. Although improved food security is positively associated with academic performance, racial disparities are evident in the academic benefits of food insecurity: Among 11th and 12th graders, white students, in comparison to black and Middle Eastern peers, receive greater academic enhancement with increased food security, while East Asian students maintained higher grades and had higher rates of attending university, even when food insecure^(10)^.

As educational attainment is an important determinant of health, one academic factor worth studying is school absenteeism. School absenteeism is associated with several short- and long-term negative outcomes in youth, including conduct disorders, mental health issues^(11)^, poor school performance and test scores^(12,13)^ and school dropout^(14)^. As with food insecurity, poverty and family variables are also relevant indicators for chronic school absenteeism^(14,15)^, and of these factors, poverty and its downstream effects are the strongest indicator of chronic school absenteeism^(16,17)^.

While both food insecurity and absenteeism negatively impact child health and well-being, little is known about the relationship between food insecurity and absenteeism, especially about this relationship in the USA. Jyoti and colleagues examined academic outcomes, but not absenteeism specifically^(8)^, and Murphy and colleagues found that “hungry” children were absent more often than “not-hungry” children^(9)^. In a systematic review and secondary data analysis examining only low-income countries, Tamiru and Belachew concluded that “students from food secure households were 57 % less likely to be absent from school … than students from food insecure households”^(16)^. Within the USA, Nevada, specifically, had child food insecurity rates above the national average (20 % *v*. 17·0 %)^(17)^ before the impacts of COVID-19, and the estimated 2020 rate of child food insecurity in Nevada was a staggering 32·9 %^(18)^. Nevada is among the states most burdened with higher per capita healthcare costs associated with food insecurity^(19)^ and ranks in the top 10 states with the highest rates of chronic absenteeism^(20)^. Therefore, we sought to determine whether household food insecurity is associated with school-level absenteeism in public elementary schools of Clark County School District in Southern Nevada.

## Methods

### Setting

Clark County School District (CCSD) is a mostly urban, racially and ethnically diverse school district located in Southern Nevada. Serving over 320 000 children, CCSD educates 75 % of students in Nevada and is the 5th largest school district in the nation. In the 2016–2017 school year, CCSD operated 216 elementary schools, increasing to 223 in the 2017–2018 school year^(21)^ and adding an addition of 10 magnet elementary schools^(22)^.

School-level variables were obtained from the 2016–2017 to 2017–2018 CCSD Accountability Reports for each elementary school in the District. The dependent variable, average daily attendance, is defined as “the percentage of the school enrollment in attendance on an ‘average school day’ as of the 100th day of school”^(21)^. Additional variables that are known risk factors for school absenteeism were also included, including demographic and student information for each school such as student race/ethnicity, gender, disability status, English language proficiency, free and reduced-price lunch eligibility and parent–teacher conference participation. Demographic and student information are provided by the Nevada Department of Education on count day. Race/ethnicity and child gender data are reported by the parent or guardian of the child during school enrollment. Students with a disability have an Individualized Education Program (IEP)^(23)^. English Language Learners are defined as students “whose: (1) primary language is not English; (2) proficiency in English is below the average proficiency of pupils at the same age or grade level whose primary language is English and (3) probability of success in a classroom in which courses of study are taught only in English is impaired because of his or her limited proficiency in English”^(24)^. Students are eligible for free or reduced price lunch (FRL) based on income eligibility: household incomes at or below 130 % of the federal poverty level qualify for free meals; household incomes between 130 and 185 % of the federal poverty level qualify for reduced price meals and “all children in households receiving benefits from the Supplemental Nutrition Assistance Program, the Food Distribution Program on Indian Reservations or Temporary Assistance for Needy Families”, foster children, those participating the Head Start program, and homeless, runaway or migrant children are eligible for free school meals^(25)^. Parent–teacher conference participation is denoted based on whether the teacher conferred with the parent or guardian in person, by telephone or using an alternative remote method.

Household-level food insecurity rates for each census tract in Clark County, NV, were provided by Three Square Food Bank and were calculated by Gundersen and colleagues as part of a Map the Meal Gap report for Feeding America^(26)^. Food insecurity rates are first estimated at the state level using established determinants of food insecurity, controlling for unobservable state and year fixed effects. The state-level coefficients are then used in combination with the same established determinants at the census tract level. A detailed methodology can be found in Gundersen *et al.* ^(26)^. Table 1 summarises CCSD average daily attendance, food insecurity and related risk factors.

**Table 1:** Summary statistics of with average daily attendance and risk factors in Clark County School District (CCSD) elementary schools for the 2016–2017 and 2017–2018 school years (*n* 223)

Variables	Mean	sd	Min	Q1	Median	Q3	Max
Attendance % (Missing = 0)	95·01	1·14	84·25	94·65	95·10	95·50	100·00
Food insecurity* (Missing = 36)	14·38	4·48	7·75	11·26	13·68	16·39	31·94
PT conference participation % (Missing = 1)	90·48	16·40	0·00	93·00	96·50	98·50	100·00
FRL eligibility % (Missing = 0)	77·92	25·21	10·92	59·72	88·55	100·00	100·00
ELL % (Missing = 5)	23·17	15·15	0·00	10·11	20·48	34·65	58·58
IEP % (Missing = 3)	14·54	8·63	5·89	12·14	13·63	15·46	100·00
Male % (Missing = 1)	52·29	3·91	39·04	50·91	52·06	53·00	85·94
White % (Missing = 2)	25·17	17·98	2·41	9·92	22·11	37·05	84·38

PT, parent/teacher; FRL, free and reduced price lunch; ELL, English language learner; IEP, individualised education plan (represents students with a disability).

* Food insecurity = average of household food insecurity rates for census tracts contained in each school catchment area.

Due to the interest in the estimated household food insecurity rates for each school catchment area, the ESRI geographic information system (GIS) mapping tool ArcMap was used to obtain an average food insecurity rate for each catchment area. To accomplish this, a raster map of estimated household food insecurity rates for each census tract was created by spatially joining the estimated food insecurity rates with the U.S. Census Bureau’s Tiger/Line shapefile^(27)^. Since census tract polygons include open spaces (parks, parking lots and roads), the resulting food insecurity pixels of such areas were removed. For this purpose, a buildup land cover map of Clark County, NV, was created by classifying a Landsat Thematic Mapper remote sensing image into urban buildup and non-buildup principle surface components. The buildup components reflect building structures representing area of the census tract with population dwellings. Because census tracts are smaller than school catchment areas, zonal statistics were computed to determine the estimated household food insecurity rate for each school catchment area by averaging the rate for each tract within the catchment area. Computationally, tracts’ food insecurity values were weighted by the area of overlap with the given catchment.

### Statistical analysis

The tobit regression model^(28)^ was applied to evaluate whether household food insecurity rate, male student percentage, white student percentage, IEP percentage, English Language Learners percentage, FRL eligibility percentage and parent–teacher conference participation rate were associated with the average daily attendance rate. The rationale to use this modelling approach is due to the fact that the dependent variable (i.e. average daily attendance rate) is a percentage variable bounded by an upper limit of 100 %, while the linear regression model is only suitable for an unbounded dependent variable^(29)^. Tobit regression coefficients can be explained similarly to ordinary least square regression coefficients, although the linear association is on the uncensored latent variable, not the observed outcome^(30)^. Model diagnostics indicated that there was no violation of normality in its residuals (normality test *P*-value = 0·2363) and variance homogeneity (White test *P*-value = 0·2423). Predictors did not have multicollinearity (all tolerances > 0·1). Data cleaning, management and analyses were conducted using SAS v9.4^(31)^. The significance level was set at 5 %, and a two-tailed test was utilised.

## Results

A total of 223 elementary schools were included in the analysis. The overall average daily attendance rate was 95·01 % (sd = 1·14). The quartile shows that the distribution was more likely positively skewed in most risk factors (the range of skewness: 0·52–8·94), except for the parent–teacher conference participation (skewness = −2·55) and FRL eligibility (skewness = −0·90). Missing data were mainly found in food insecurity rates. When including all variables in the same model, the valid final sample size was reduced to 185. Table 2 shows that the average daily attendance rate was negatively associated with all risk factors, except for the parent–teacher conference participation (*P* = 0·18) and white student percentage (*P* = 0·28). Strong correlations among risk factors only appeared between the IEP and male student percentage (*P* = 0·85) and between the English Language Learners and white student percentage (*P* = −0·80).

**Table 2:** Correlation matrix among average daily attendance and risk factors in Clark County School District (CCSD) elementary schools for the 2016–2017 and 2017–2018 school years (*n* 223)

Variables	Attendance	Food insecurity	PT conference participation	FRL eligible	ELL	IEP	Male	White
Attendance	1·00							
Food insecurity^*^	−0·33	1·00						
PT conference participation %	0·18	−0·05	1·00					
FRL eligibility %	−0·40	0·35	−0·05	1·00				
ELL %	−0·12	0·28	−0·06	0·60	1·00			
IEP %	−0·65	0·11	−0·12	0·13	−0·17	1·00		
Male %	−0·54	0·07	−0·15	0·04	−0·14	0·85	1·00	
White %	0·28	−0·37	0·01	−0·61	−0·80	0·12	0·19	1·00

PT, parent/teacher; FRL, free and reduced price lunch; ELL, English language learner; IEP, individualised education plan (represents students with a disability).

* Food insecurity = average of household food insecurity rates for census tracts contained in each school catchment area.

Table 3 shows that the predicted average daily attendance rate significantly decreased by −0·0232 % (*P*-value = 0·022; 95 % CI = −0·043, −0·003) per 1 % increase in the food insecurity rate. Moreover, the daily attendance rate was also negatively associated with FRL eligibility, IEP and male student percentage. In particular, compared with the other risk factors, the IEP student percentage resulted in the largest association on daily attendance rate by −0·039 % (*P*-value = 0·033; 95 % CI = −0·076, −0·003). A significant positive association with daily attendance rate was only found in the parent–teacher conference participation rate and the white student percentage, where a 1 % increase significantly elevated the predicted average daily attendance rate by 0·006 % (*P*-value = 0·025; 95 % CI = 0·001, 0·011) and 0·011 % (*P*-value = 0·022; 95 % CI = 0·002, 0·020), respectively.

**Table 3:** Tobit regression model results showing variable associations with average daily attendance in Clark County School District (CCSD) elementary schools for the 2016–2017 and 2017–2018 school years (*n* 185)

	Estimate	95 % CI	*P*-value
Food insecurity*	−0·023	−0·043, −0·003	0·022
PT conference participation %	0·006	0·001, 0·011	0·025
FRL eligibility %	−0·010	−0·015, −0·005	< 0·001
ELL %	0·008	−0·022, 0·018	0·129
IEP %	−0·039	−0·076, −0·003	0·033
Male %	−0·004	−0·048, 0·039	0·842
White %	0·011	0·002, 0·020	0·022

PT, parent/teacher; FRL, free and reduced price lunch; ELL, English language learner; IEP, individualised education plan (represents students with a disability).

* Food insecurity = average of household food insecurity rates for census tracts contained in each school catchment area.

## Discussion

Food insecurity is a critical factor for child health, development and well-being^(1)^. Existing research suggests that for children, food insecurity is associated with adverse childhood behaviour, emotional development and academic performance, including school absenteeism^(1,7,9)^. School absenteeism is also associated with negative behavioural health and academic indicators^(11–14)^. However, not much is yet known about the intersection of food insecurity and school absenteeism, particularly in higher income countries. Our Southern Nevada-focused case study aims to reduce this knowledge gap. Using school-level data from one of the largest school districts in the USA and corresponding household-level food insecurity data for each school catchment area, the current study examined the relationship between the average daily school attendance rate and estimated food insecurity rate by applying spatial and statistical analysis and accounting for other factors related to school attendance. The finding that the average daily attendance rate was significantly lower with higher rates of food insecurity is important: it establishes a link between these two factors, both of which are critical for childhood health, education and well-being.

Average daily attendance rates were lower for schools with school catchment areas that had higher average household food insecurity rates in the current study. This finding is similar to that of Murphy and colleagues^(9)^, who found that “hungry” children were more likely to be absent. Food insecurity is associated with numerous physical and mental health outcomes in children and these outcomes, such as internalising or externalising behaviours, may influence a child’s likelihood to attend school^(32)^. Additionally, while many food insecure households try to shield their children from experiencing the effects of food insecurity^(33)^, stress associated with difficultly or inability to provide basic necessities may result in caregiver distress and negative health outcomes that may influence caregiver ability to foster healthy school-related behaviours, such as school attendance^(32)^. From an interventional perspective, this is concerning, as schools often provide entry points for resources that may help to mitigate some of the effects of food insecurity. For example, the National School Lunch Program is an income-based entitlement programme that provides meals that meet certain nutritional requirements, and, for students, who qualify based on family income, these meals are provided for free or at a reduced price^(34)^. It has been found that food insecure children are more likely to eat school meals and receive more of their nutrient intake from school meals than food secure children^(34)^. When food insecure children miss more days of school, it is possible that the opportunities for school-based intervention may be more limited. The goal of the National School Lunch Program has always been to ensure children’s physical needs are met through the requisition of a nutritious meal so they can attend school and be prepared to receive an adequate education. That is, preventing absenteeism has always been a goal of the National School Lunch Program. Thus, if food insecurity prevents children from going to school, it undermines both goals of the school meals programme, and bolstering attendance may be especially important.

Schools with greater rates of children with documented disabilities (i.e. IEP) also had lower rates of daily attendance. This finding is similar to those of other studies^(35)^, including a study using data from the nationally representative National Health Interview Survey, which found a dose–response relationship between the number of developmental disabilities and the odds of chronic absenteeism^(36)^. This is concerning, however, because children with disabilities are likely in need of the specialised services that are associated with having an IEP, so being absent from school would limit the ability to provide such services. Interruption in or inability to provide comprehensive services due to absenteeism may impact their health and educational outcomes. Additionally, we found that schools with higher rates of children who are eligible for FRL had lower rates of daily attendance. Other studies have found correlations between income or poverty and absenteeism^(14,15,37)^. However, by nature of being an income-based entitlement programme, the National School Lunch Program serves children from lower income families. As discussed, poverty is also a critical indicator for chronic school absenteeism^(14,15,37,38)^. It is therefore possible that many children eligible for the National School Lunch Program are also more likely to miss more days of school and not be receiving the full benefits of programme participation. This is not to suggest that the size or scope of the National School Lunch Program should be reduced. Rather, it suggests that promoting school attendance by children who are eligible for the National School Lunch Program may be doubly beneficial.

As the findings of the current study indicate, other factors may be at play to enhance attendance and reduce absenteeism. The finding that average daily attendance rates increased significantly with increased percentages of white students in a school catchment area is consistent with existing literature, which indicates that non-white students experience school absenteeism at disproportionate rates compared with white students and that relative social advantage plays a role^(39)^. This finding reaffirms the need to prioritise absenteeism interventions, including policies and programmes that prioritise groups such as racial and ethnic minorities that experience inequity. Similarly, the finding that average daily attendance rates increased significantly with increased parent–teacher conference participation rates is consistent with a review by Kearney^(14)^, which found that limited parental involvement (e.g. attendance at parent–teacher conferences) is a risk factor for chronic school absenteeism. This finding supports efforts to engage parents in the school and in children’s learning through mechanisms like parent–teacher conferences.

The current study is not without limitations. The current analysis solely examined public elementary schools in CCSD, while CCSD is a large and diverse school district located in Southern Nevada, findings cannot be generalised outside of the given sample. Additionally, given the nature of a secondary analysis, school-level variables rather than individual-level absenteeism rates and risk factors were obtained and used in the analysis. The sample size was large, bolstering the decision to use this approach. However, to circumvent ecological correlations, the current study would have needed individual-level data, which was not available for the current analysis. Individual data would have provided a much larger sample and may have more closely approximated the population. Many variables known to be associated with missed school and absenteeism were included in the current analysis. But given the complex nature of the outcome, interpretation of these results should note that other factors that may have a correlational relationship and were not included in the analysis may be relevant. For example, the current analysis was unable to acquire school climate data or data on homelessness, factors known to correlate with absenteeism^(14)^. Household-level food insecurity rates were not available at the school level, so spatial analysis through GIS was used to obtain the average rate of food insecurity for each school catchment area from the census tract level, which may introduce some level of spatial error. Additionally, the household-level food insecurity rate for each census tract is based off of a state-level estimate^(28)^, though these estimates have been found to be robust and correlate with primary data^(40)^, the estimates are subject to measurement errors in their own right. Finally, household food insecurity estimates do not consider if children are present in the home or child-specific food insecurity.

Despite these limitations, findings from the current study may have practical implications for how to promote school attendance through interventions that rely on policies, programmes and practices to help schools and communities support students beyond the classroom. Specifically, schools, communities and decision makers should continue to recognise the role of food insecurity in impacting academic indicators, including attendance. Interventions that aim to reduce food insecurity of students’ households may also pay off in increased student attendance and, down the line, better educational attainment, development and health. For students in food insecure households, participation (not just eligibility) in the National School Lunch Program may be a way to dampen the overall household food insecurity by ensuring that students have access to meals when in school. This too has the potential to yield attendance dividends. The connection between the percentage of students with IEPs and reduced daily attendance rates also suggests that targeted efforts to support students with IEPs may simultaneously increase school attendance. Meanwhile, the lower rates of attendance at schools with greater percentages of non-white students confirm that interventions focused on schools with greater percentages of students who experience racial and ethnic disparities may support both district-level attendance efforts and outcomes for children who are disproportionately vulnerable to absenteeism. Finally, the positive role of parent–teacher conferences, a proxy for parental participation in formal schooling, suggests that efforts to bolster parental participation too may be a way to increase school attendance.
